# Polydatin promotes the neuronal differentiation of bone marrow mesenchymal stem cells in vitro and in vivo: Involvement of Nrf2 signalling pathway

**DOI:** 10.1111/jcmm.15187

**Published:** 2020-04-16

**Authors:** Jiheng Zhan, Xing Li, Dan Luo, Yu Hou, Yonghui Hou, Shudong Chen, Zhifeng Xiao, Jiyao Luan, Dingkun Lin

**Affiliations:** ^1^ Second Clinical College Guangzhou University of Chinese Medicine Guangzhou China; ^2^ Lingnan Medical Research Center Guangzhou University of Chinese Medicine Guangzhou China; ^3^ Department of Spine Surgery The Second Affiliated Hospital of Guangzhou University of Chinese Medicine Guangzhou China

**Keywords:** bone marrow mesenchymal stem cells, functional recovery, neuronal differentiation, nuclear factor E2–related factor 2, polydatin, spinal cord injury

## Abstract

Bone marrow mesenchymal stem cell (BMSC) transplantation represents a promising repair strategy following spinal cord injury (SCI), although the therapeutic effects are minimal due to their limited neural differentiation potential. Polydatin (PD), a key component of the Chinese herb *Polygonum cuspidatum*, exerts significant neuroprotective effects in various central nervous system disorders and protects BMSCs against oxidative injury. However, the effect of PD on the neuronal differentiation of BMSCs, and the underlying mechanisms remain inadequately understood. In this study, we induced neuronal differentiation of BMSCs in the presence of PD, and analysed the Nrf2 signalling and neuronal differentiation markers using routine molecular assays. We also established an in vivo model of SCI and assessed the locomotor function of the mice through hindlimb movements and electrophysiological measurements. Finally, tissue regeneration was evaluated by H&E staining, Nissl staining and transmission electron microscopy. PD (30 μmol/L) markedly facilitated BMSC differentiation into neuron‐like cells by activating the Nrf2 pathway and increased the expression of neuronal markers in the transplanted BMSCs at the injured spinal cord sites. Furthermore, compared with either monotherapy, the combination of PD and BMSC transplantation promoted axonal rehabilitation, attenuated glial scar formation and promoted axonal generation across the glial scar, thereby enhancing recovery of hindlimb locomotor function. Taken together, PD augments the neuronal differentiation of BMSCs via Nrf2 activation and improves functional recovery, indicating a promising new therapeutic approach against SCI.

## 1 INTRODUCTION

Spinal cord injury (SCI) is a devastating central nervous system (CNS) trauma that results in catastrophic dysfunction, high disability rate and huge cost for the patient.^1^ Neuronal apoptosis, axonotmesis, demyelination and oligodendrocyte destruction are the direct causes of spinal cord dysfunction.^2^ Poor neuronal activity, glial scar formation, inhibited axon growth and an inflammatory environment also hinder nerve regeneration in the injured spinal cord.^3^ The surviving axons become less efficient as the disease progresses, and the demyelinated axons fail to transmit sensory or motor nerve impulses.^4^, ^5^ Although there are no fully restorative treatments for SCI, various cellular and molecular therapies have exhibited promising results in animal models,^6^, ^7^, ^8^ gradually changing the public's pessimistic attitude towards SCI.

**FIGURE 1:**
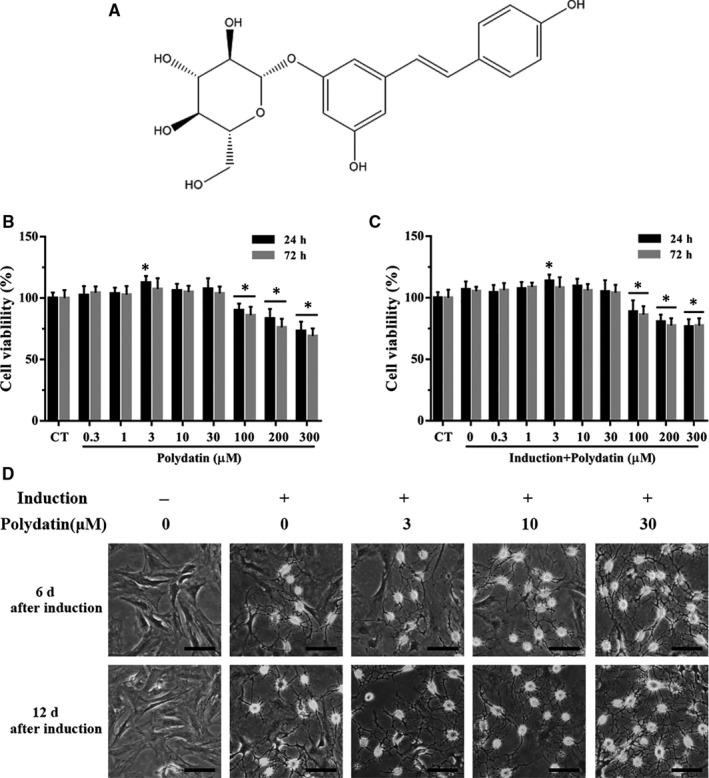
Effects of PD on BMSC morphology and neural differentiation potential. A, The structure of polydatin (PD). B, Viability of BMSCs with/out PD treatment after 24 and 72 h of culture. C, Viability of BMSCs treated with varying concentrations of PD after 24‐ and 72‐h neural induction. D, Morphological changes in the BMSCs after 6‐ and 12‐day neural induction. Scale bar = 100 μm. *P < .05, significant difference vs the CT

Recently, stem cell–based therapy, especially with the use of bone marrow mesenchymal stem cells (BMSCs), has been shown to be a promising therapy for SCI. BMSCs are easily isolated and amplified, with strong self‐renewal and multipotent differentiation capacity, as well as low immunogenicity.^9^ However, only a small fraction of grafted BMSCs successfully differentiate into neuron‐like cells in vivo, which could perhaps be the key to successful treatment for SCI. Therefore, an effective approach aimed at enhancing the neural differentiation ability of BMSCs is urgently needed. Polydatin (PD, Figure 1A), a glucoside of resveratrol, is an active ingredient isolated from the dried rhizome of *Polygonum cuspidatum*. Emerging studies have demonstrated that PD may alleviate secondary injury after SCI by suppressing oxidative stress and microglia apoptosis.^10^, ^11^, ^12^ In addition, our previous studies proved that PD can facilitate BMSC migration and protect BMSCs from oxidative stress‐induced apoptosis.^13^, ^14^ Together, the studies above suggest that PD could be used in combination with BMSC transplantation for the treatment of SCI. However, it remains largely unclear whether PD promotes neural differentiation of transplanted BMSCs.

Nuclear factor E2–related factor 2 (Nrf2), a member of the Cap‐n‐collar (CNC) regulatory protein family, is activated in response to oxidative stress to initiate the transcription of downstream genes whose function is to enhance the resistance to oxidative injury.^15^, ^16^, ^17^ Additionally, the Nrf2/antioxidant response element (ARE) cascade is a well‐studied signalling pathway that is closely related to the prevention of neuronal apoptosis.^18^ As Nrf2 plays a key role in the regulation of neuronal differentiation,^19^, ^20^ we suggested that PD may affect neuronal differentiation of BMSCs via the Nrf2 pathway. In order to gain insight into this issue, we explored it here through cell experiments and SCI animal models. Our findings demonstrate for the first time that PD can promote the differentiation of BMSCs into neuron‐like cells in vitro and in vivo, thereby improving behavioural outcome, which is closely related to the activation of the Nrf2 pathway.

## 2 MATERIALS AND METHODS

### 2.1 Usage of animals and ethics statement

Healthy adult male C57BL/6 mice were obtained from the Guangdong Medical Experimental Animal Center (Foshan, China, Certificate No. 44007200047868) and housed in a strictly controlled environment condition with free access to food and water. All animal experiments were approved by the Institutional Animal Care and Use Committee of Guangzhou University of Chinese Medicine, and performed in accordance with the ‘NIH Guide for the Care and Use of Laboratory Animals’.

### 2.2 Isolation and culture of BMSCs

Bone marrow mesenchymal stem cells were isolated by the whole bone marrow adherence method with minor modifications.^21^ The cells were cultured in Dulbecco's modified Eagle's medium (DMEM) supplemented with 15% fetal bovine serum (FBS), 2 mmol/L L‐glutamine, 100 units/mL penicillin and 100 μg/mL streptomycin under a 5% CO_2_ atmosphere at 37°C, and harvested at subconfluency using 0.25% trypsin‐EDTA. The multipotent differentiation capacity of the isolated cells was determined using the osteogenic differentiation kit (Cyagen) and chondrogenic differentiation kit (Cyagen) as previously described.^22^ The BMSCs were also characterized by flow cytometry using a mesenchymal stem cell surface marker detection kit (Cyagen). All BMSCs used throughout this study were between passages 2 and 4.

### 2.3 Neuronal induction of BMSCs

Bone marrow mesenchymal stem cells were cultured in a neuronal induction medium (NIM) consisting of DMEM supplemented with 2% N2, 2% B27 (Gibco), 25 ng/mL bFGF, 25 ng/mL BDNF and 40 ng/mL NGF (PeproTech) with or without different concentrations (0.3‐300 μmol/L) of PD (Sigma‐Aldrich; 0.1% working solution in DMSO; Figure S1A). To further confirm that Nrf2 signalling is involved in PD‐induced neural differentiation, cells were grown in the presence/absence of brusatol (100 nmol/L; Chengdu Herbpurify) as appropriate. Morphologic changes were observed under a phase‐contrast microscopy (Leica).

### 2.4 Cell viability analysis

Cell viability was determined using the Cell Counting Kit‐8 (CCK‐8) assay. Briefly, BMSCs were seeded into 96‐well plates (1 × 10^4^ cell/well) and cultured for 24 hours. The medium was then replaced with fresh medium with or without PD, and cultured for varying durations. At each time‐point, 10 μL CCK‐8 solution (KeyGEN, China) was added per well, and the cells were further incubated for 2 hours at 37℃. The absorbance of each well at 450 nm was measured using a micro‐plate reader (Bio‐Rad).

### 2.5 Cellular immunofluorescence

The induced cells on glass coverslips were fixed in 4% paraformaldehyde (PFA), permeabilized with 0.3% Triton X‐100 and blocked with 5% normal goat serum in PBS. Following overnight incubation at 4°C with primary antibodies against BrdU (1:200, CST), NF‐M (1:200, Abcam), microtubule‐associated protein 2 (MAP‐2, 1:100, CST), ChAT (1:200, Abcam) and NeuN (1:300, Abcam), cells were then washed thrice with PBS and subsequently incubated with fluorescein‐conjugated secondary antibodies. After several washes with PBS, the coverslips were mounted and observed under a fluorescence microscope (Leica).

### 2.6 Establishment of the SCI model and treatment regimen

A total of 150 mice (20‐25 g) were randomly divided into the sham‐operated, SCI, PD‐treated, BMSC‐treated and PD+BMSC groups (N = 30 each; Figure S1B). Each animal was anaesthetized intraperitoneally with 2% (w/v) pentobarbital sodium (40 mg/kg), and the spinal cord was exposed by laminectomy at the T9‐T10 vertebral level. A contusion simulating thoracic SCI was produced using a pneumatic impact device in accordance with Allen's method.^23^ The impact velocity was set at 0.5 m/s. The depth and duration of the impact were kept constant at 0.6 mm and at 80 ms, respectively. Postoperatively, the animals’ urinary bladders were manually voided twice daily. In the sham‐operated group, each mouse underwent a laminectomy, with no contusion injury performed. In the remaining groups, the mice were gastrically perfused with PD (20 mg/kg) once a day and/or transplanted with BMSCs at 5 days post‐injury (dpi) as appropriate. The surgical wound was opened, and a 3 μL suspension containing 2 × 10^5^ BrdU‐labelled BMSCs was injected into the injured site using a Hamilton syringe (Hamilton). The tip of the micropipette was kept in the spinal cord for 5 minutes after the injection. The mice in the SCI and PD groups were similarly injected with sterile PBS. After treatment, mice were killed and the spinal cords were extracted and stored at −80°C for subsequent experiments.

### 2.7 Western blotting

From the cellular/tissue homogenates, the protein concentration was determined using a BCA assay kit (Beyotime). Equal amounts of total protein were separated on SDS‐PAGE gels and transferred onto PVDF membranes. After blocking with 5% skim milk at room temperature, the membranes were incubated overnight with the primary antibody solutions at 4°C. The membranes were then rinsed thrice with TBST, followed by 1‐hour incubation with horseradish peroxidase (HRP)‐conjugated secondary antibodies. All signals were visualized by a ChemiDoc XRS+Imaging System (Bio‐Rad), and the results were quantified with ImageJ software (NIH).

### 2.8 RNA extraction and qRT‐PCR

After treatment, the mRNA was isolated from the cultured cells or spinal cord tissues using the MiniBEST Universal RNA Extraction Kit (Takara, Japan) and cDNA was synthesized. The qRT‐PCR was performed in a CFX96™ Real‐Time PCR Detection System (Bio‐Rad) using SYBR Green PCR Mix with the following conditions: initial denaturation at 95℃ for 2 minutes, followed by 40 cycles of denaturation at 95°C for 5 seconds; annealing at 60°C for 30 seconds; and extension at 72°C for 45 seconds. The relative mRNA levels were analysed using the 2^−ΔΔCt^ method and normalized to those of the internal control *β*‐actin. All primer sequences are listed in Table S1.

### 2.9 Histopathology and tissue immunofluorescence

The spinal cord tissues were fixed in 10% formaldehyde, embedded in paraffin and cut into 5‐μm‐thick transverse sections. Hematoxylin and eosin (H&E) staining and Nissl staining were performed according to the manufacturer's instructions. For immunofluorescence, 10‐μm‐thick transverse frozen sections were incubated with primary antibodies targeting MAP‐2 (1:200) and Nrf2 (1:200, R&D systems), and the longitudinal sections, with antibodies against GFAP (1:500, Abcam), NF‐200 (1:200, Boster), Tuj1 (1:200, CST), GAP43 (1:300, Novus), BrdU (1:200), Nestin (1:200, Novus), NeuN (1:300) and NSE (1:100, Abcam). After incubation with species‐specific secondary antibodies conjugated with Alexa Fluor 488 (1:300, CST) or Cyanine3 (1:200, Invitrogen) for 1 hour at room temperature, the sections were counterstained with 4′,6‐diamidino‐2‐phenylindole (DAPI) and observed under a laser‐scanning confocal microscope (Leica).

### 2.10 Transmission electron microscopy (TEM)

The specimens were fixed in 2.5% glutaraldehyde at 4°C for 120 minutes, post‐fixed in 2% buffered osmium tetroxide and blocked with 2% uranyl acetate. The processed tissues were dehydrated in a mixture of ethanol and acetone, and then embedded in Epon‐Araldite. Semi‐thin sections were cut and stained with toluidine blue to observe the appropriate location. Finally, ultrathin sections were stained with lead citrate. All sections were examined with a JEM‐1200EX electron microscope (JEOL).

### 2.11 Assessment of locomotor function

Functional recovery was evaluated using the Basso‐Beattie‐Bresnahan (BBB) locomotor rating scale and inclined plane test at different time‐points post‐injury. The BBB scale (0 = complete paralysis to 21 = normal gait) was graded on the basis of joint movements, gait co‐ordination and weight support. The inclined plane test was performed to assess postural stability. The tests were conducted by two independent examiners who were blinded to the experimental protocols.

### 2.12 Spinal cord evoked potential (SCEP)

The technique used to evoke SCEP has been well described in mammals following spinal trauma, due to its relative ease of use and high reliability. In the present study, SCEP values were assessed 4 weeks after the SCI operation, according to previously described protocols.^24^ To elicit a SCEP, a pulse stimulation was transmitted through the electrodes connected to a BL‐420 biological function experiment system. One hundred SCEP responses were recorded for each animal, and the amplitudes and latent periods of SCEP signal were analysed.

### 2.13 Statistical analysis

All data were presented as mean ± standard deviation (SD) of at least three independent experiments and were analysed using SPSS 24.0 software (SPSS Inc). One‐way analysis of variance (ANOVA) and unpaired Student's *t* test were used to respectively compare multiple groups and two groups. *P*‐values < .05 were considered statistically significant.

## 3 RESULTS

### 3.1 Characterization of BMSCs

The cultured BMSCs at different passages (Figure S2A,B) and the BrdU‐labelled cells prior to transplantation (Figure S2C) were respectively observed by light and fluorescence microscopy. The osteoblast and chondrocyte‐like cells induced by the respective differentiation media were validated by suitable tests (Figure S2D‐F). Finally, flow cytometric analysis revealed that the cultured BMSCs were positive for CD29 and CD90.2 but negative for CD31 and CD117. Partial expression of CD34 was also noted (Figure S2G).

### 3.2 PD maintains BMSC viability and promotes neuronal differentiation

While BMSC viability was not affected by very low dose of PD (<3 μmol/L) even after a 72‐hours treatment, high doses (≥100 µmol/L) significantly decreased the number of viable cells in a concentration‐dependent manner (Figure 1B). In addition, 3‐30 μmol/L of PD slightly improved BMSC viability. After 24 hours of neural induction, the cells showed significantly higher proliferation, which was abrogated by high concentrations of PD (100‐300 μmol/L, Figure 1C). Accordingly, we used 3‐30 μmol/L PD for the subsequent experiments.

**FIGURE 2:**
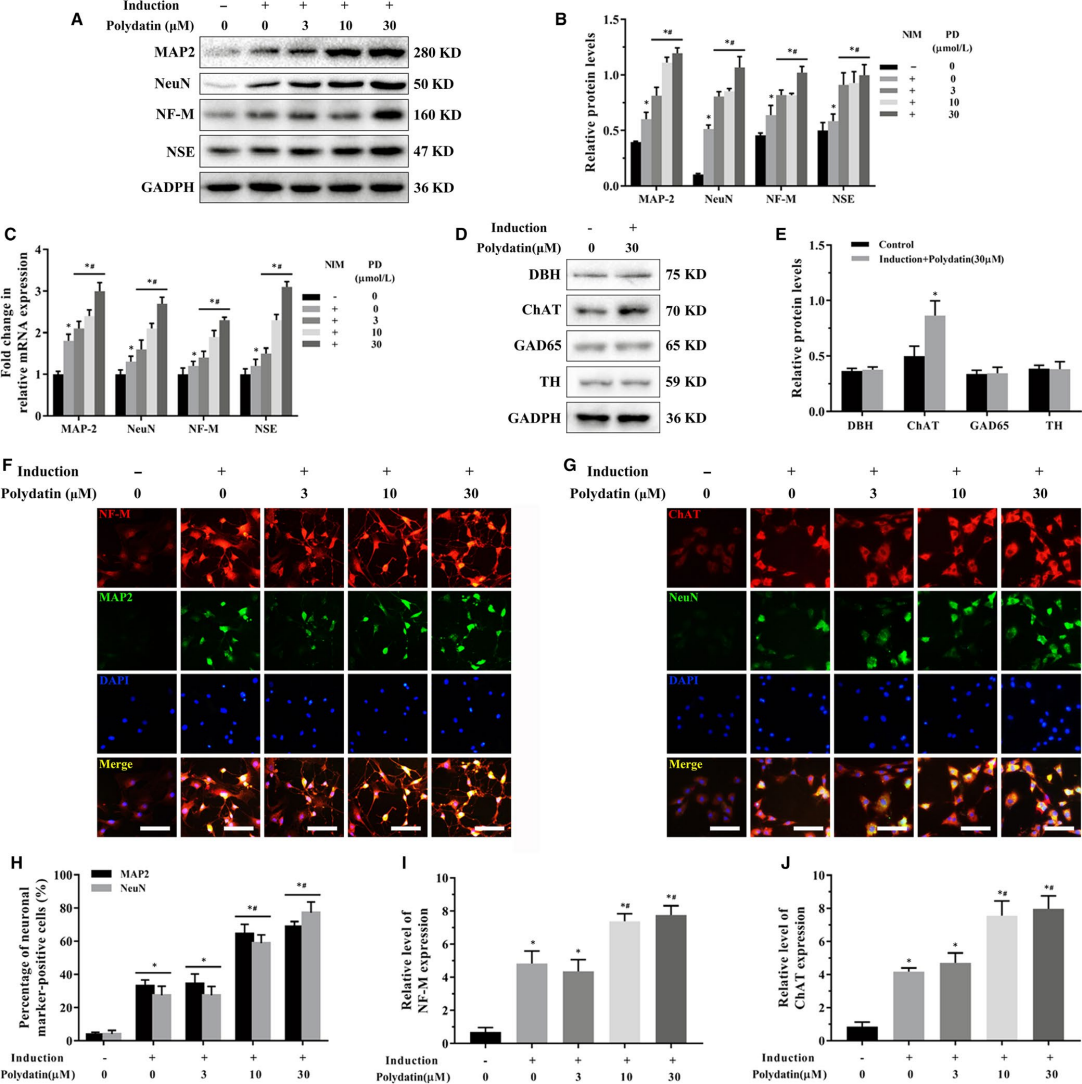
PD enhances neural differentiation of BMSCs. A, Immunoblot showing MAP‐2, NeuN, NF‐M and NSE protein levels in BMSCs after 12 days of neural induction in the presence of 0‐30 µmol/L PD. B, Quantification of the relative protein levels. C, Fold changes in neural marker mRNAs compared with the CT group. D, Immunoblot showing DBH, ChAT, GAD65 and TH levels in each group. E, Quantification of the relative protein levels. (F, G) Representative immunofluorescence images showing in situ expression of MAP‐2, NeuN, NF‐M and ChAT (scale bar = 100 μm). H‐J, The number of marker‐positive cells relative to the total BMSCs. *P < .05 vs CT; ^#^ *P* < .05 vs standard induction group

BMSCs exhibited the neuron‐like characteristic neurite outgrowth after 12‐day neural induction regardless of PD treatment (Figure 1D). However, the PD‐treated cells showed significantly higher levels of MAP‐2, NeuN, NF‐M and NSE proteins than the untreated controls in a concentration‐dependent manner (Figure 2A,B). In addition, the mRNA expression levels of these neural markers also increased twofold following exposure to 30 µmol/L PD (Figure 2C). The neuronal‐like cells induced with concurrent PD treatment also showed high levels of ChAT, a definitive marker of cholinergic neurons, compared with the standard induction group (Figure 2F–J), and also lacked GAD65, DBH and TH (Figure 2D,E). Taken together, PD promoted differentiation of BMSCs towards cholinergic neurons.

### 3.3 PD and BMSCs reduced tissue damage in the injured spinal cord

**FIGURE 3:**
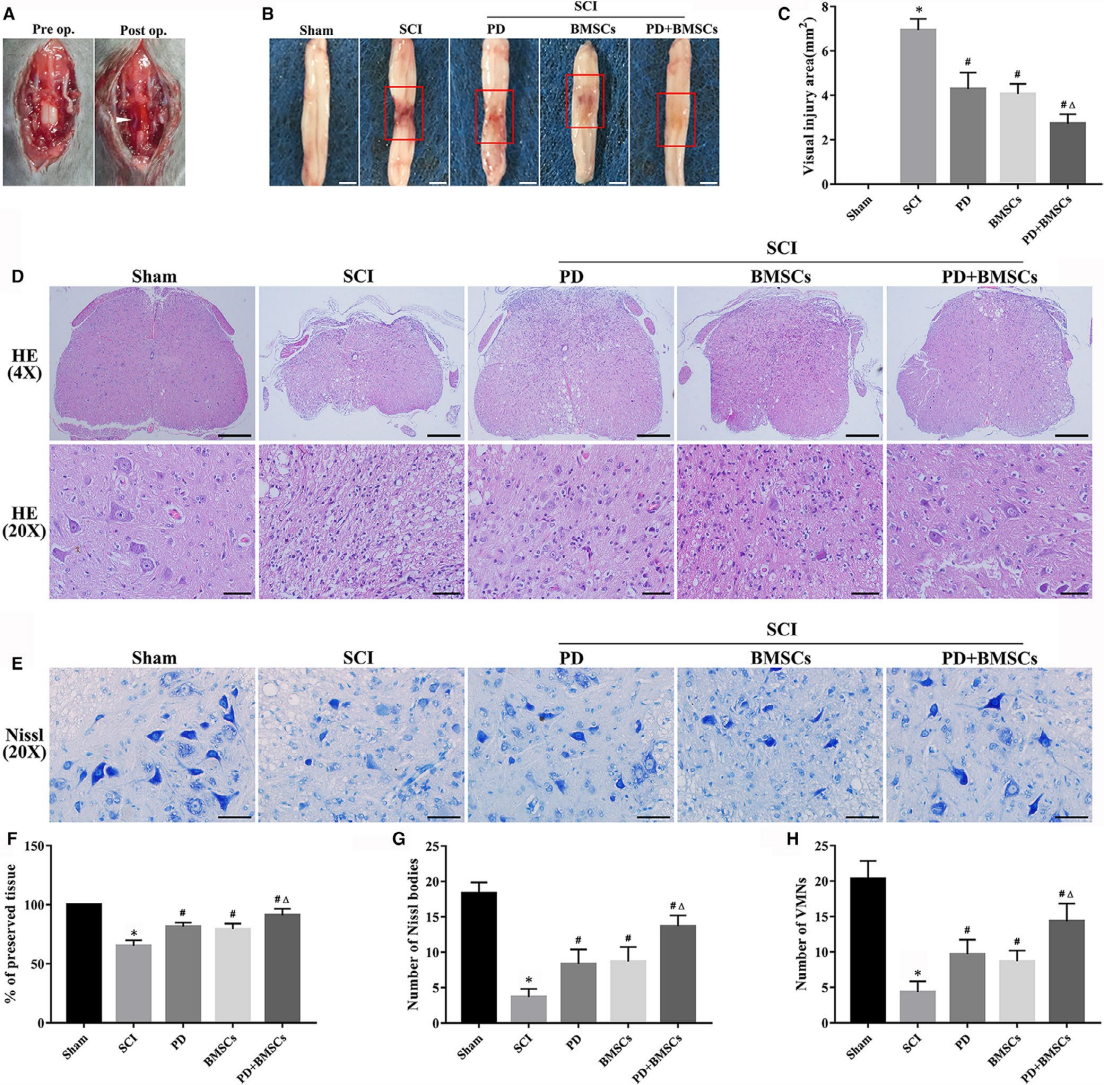
PD and BMSCs alleviated tissue damage and neuronal loss after SCI. A, Surface congestion and oedema on the spinal cord after contusion injury. B, Gross morphology of the spinal cord at 28 dpi (scale bar = 2 mm), with the reddish connective tissue (inset rectangle) as the site of SCI. C, Quantification of the lesion area. Representative images of (D) H&E staining (scale bar = 300 and 50 μm) and (E) Nissl staining (scale bar = 50 μm). F, Percentage of preserved tissue, and the number of (G) Nissl bodies and (H) VMNs in the spinal cord. *P < .05 vs sham group; ^#^ *P* < .05 vs SCI; and ^Δ^ *P* < .05 vs PD and BMSCs

Contusion injury to the spinal cord led to rapid congestion and oedema on the surface (Figure 3A). While both PD and BMSCs attenuated tissue injury, their combination resulted in the smallest lesions (Figure 3B,C). As shown in Figure 3D,F, the dorsal white matter and central grey matter of the SCI mice were severely damaged and restored to varying degrees by different treatments. Not surprisingly, the percentage of preserved tissue was higher after the combination therapy than the other treatment groups. Furthermore, PD+BMSCs also mitigated the SCI‐induced loss in Nissl bodies and ventral motor neurons (VMNs) (Figure 3E,G,H). Overall, the combination of PD and BMSC transplantation attenuated SCI‐induced neuronal damage and the histopathological damage.

### 3.4 PD promoted neural differentiation of BMSCs in the injured spinal cord

**FIGURE 4:**
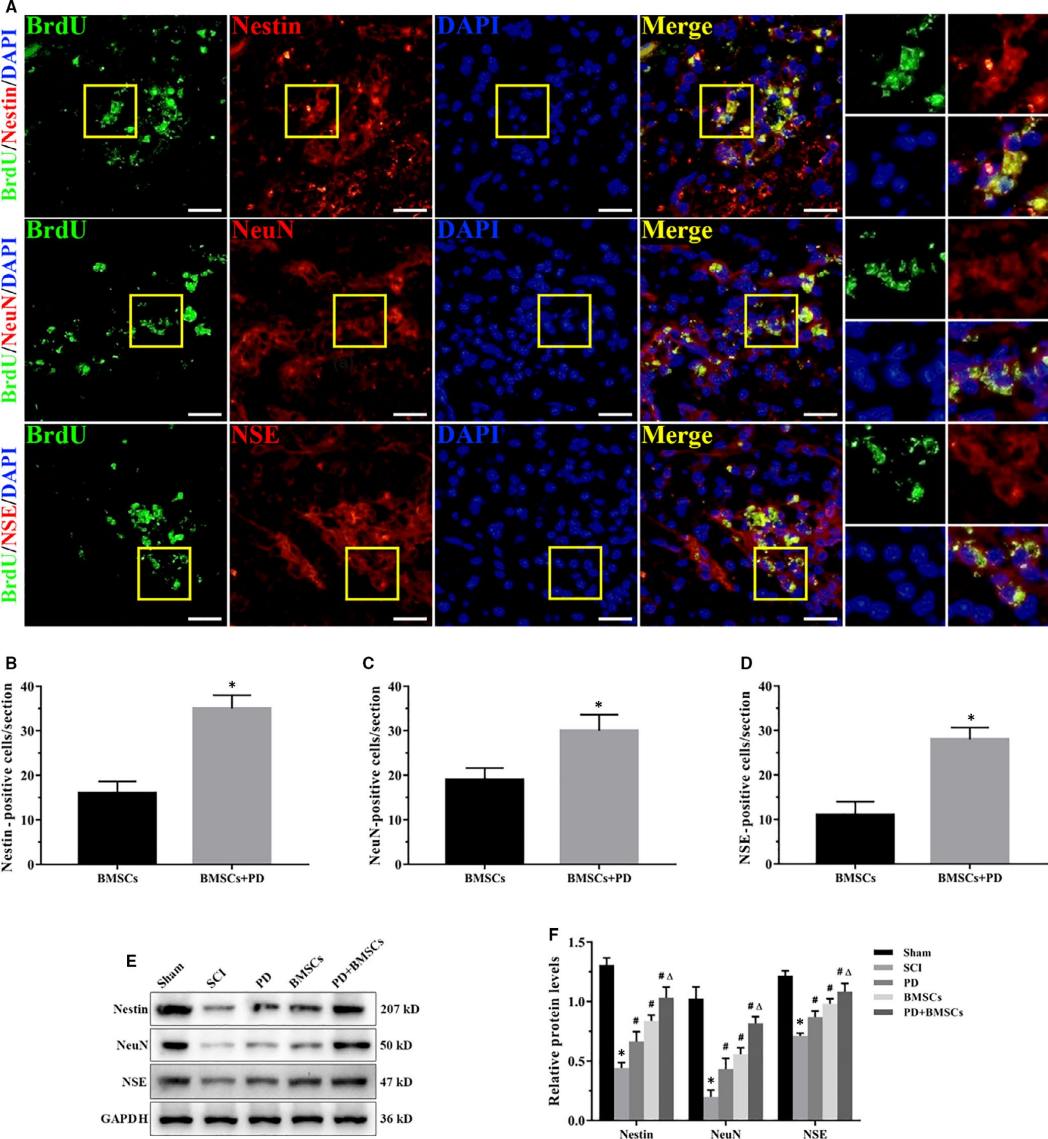
PD promoted neuronal‐like differentiation of BMSCs in the injured spinal cord. A, Immunofluorescence images of spinal cords showing neuron‐like cells in the PD + BMSC group (scale bar = 20 μm). Inset squares indicate the co‐localization of BrdU with Nestin, NeuN or NSE. B‐D, Quantitative analysis of fluorescence intensities, *P < .05 vs BMSCs. E, Immunoblot showing Nestin, NeuN and NSE levels in each group. F, Quantification of relative protein levels. *P < .05 vs sham; ^#^ *P* < .05 vs SCI; and ^Δ^ *P* < .05 vs PD and BMSCs

The transplanted BMSCs were tracked through BrdU labelling, and their differentiation at the lesion sites was evaluated via neural markers (Nestin, NeuN and NSE). The BrdU+ cells were mainly distributed around the injured site, and the number of cells co‐expressing by BrdU and Nestin, NeuN or NSE was twofold higher in the PD+BMSC group than the BMSC‐transplanted group (Figure 4A‐D, Figure S3). As shown in Figure 4E,F, the neural markers were down‐regulated after SCI and restored by PD treatment and/or BMSC transplantation. The combination of PD and BMSCs caused a more remarkable increase in their expression levels. Together, augmenting BMSC transplantation with PD significantly increased the proportion of neuron‐like cells in the spinal cord after contusion injury compared with either monotherapy.

### 3.5 PD and BMSCs promoted axonal regeneration and attenuated glial scars in the injured spinal cord

**FIGURE 5:**
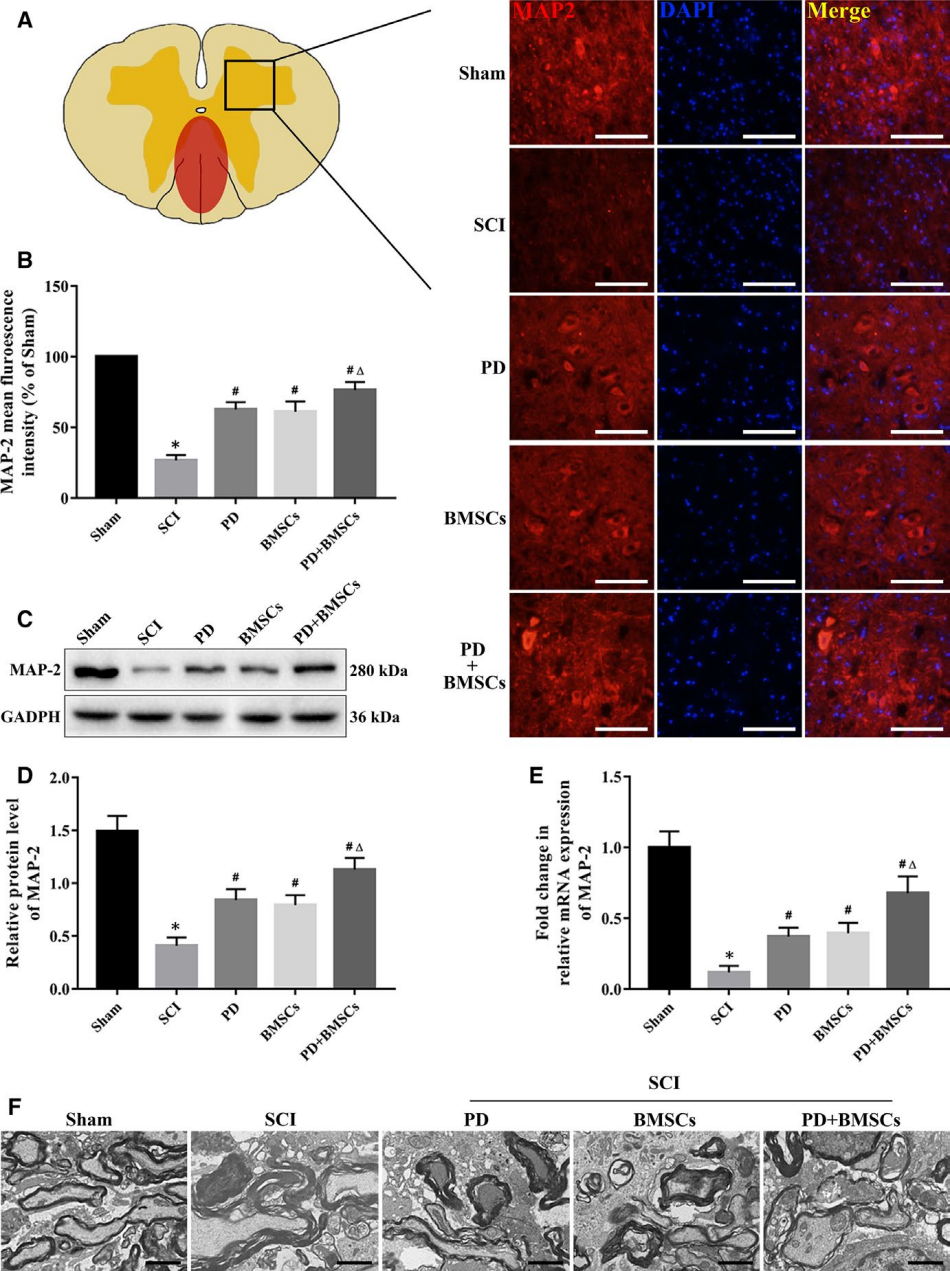
PD and BMSCs promoted axonal rehabilitation after SCI. A, Immunofluorescence images of axons expressing MAP‐2 (scale bar = 100 μm). B, Quantitative analysis of the fluorescence intensities. (C, D) Relative MAP‐2 protein levels in each group. E, Relative MAP‐2 mRNA levels in each group. F, Representative TEM images of the myelin sheath at 28 dpi (scale bar = 2 μm). *P < .05 vs sham; ^#^ *P* < .05 vs SCI; and ^Δ^ *P* < .05 vs PD and BMSCs

Axonal rehabilitation is a critical aspect of motor function and sensory recovery after SCI. Therefore, we analysed the in situ expression levels of MAP‐2, a major constituent of axon microtubules, in the spinal cord at 28 dpi. As shown in Figure 5A,B, the MAP‐2–positive axons were markedly decreased and disorganized after SCI, while the combination of PD and BMSCs restored the density and arrangement of the axons close to those in the sham group. The alterations in MAP‐2 expression levels were also confirmed by Western blotting and qRT‐PCR (Figure 5C‐E). In addition, TEM images showed vacuolation, acantholysis and loss of microtubules in the myelin sheath after injury, which were considerably restored by the combination of PD and BMSCs compared with either monotherapy (Figure 5F). Axonal regeneration was determined by evaluating the axons expressing Tuj1 and GAP43, a marker for growing axons. Most Tuj1‐positive axons were localized around the BMSC‐transplanted site in the co‐treated animals and localized with the GAP43‐positive axons, indicating that nearly half of the axons at the injured site were undergoing regeneration (Figure S4).

**FIGURE 6:**
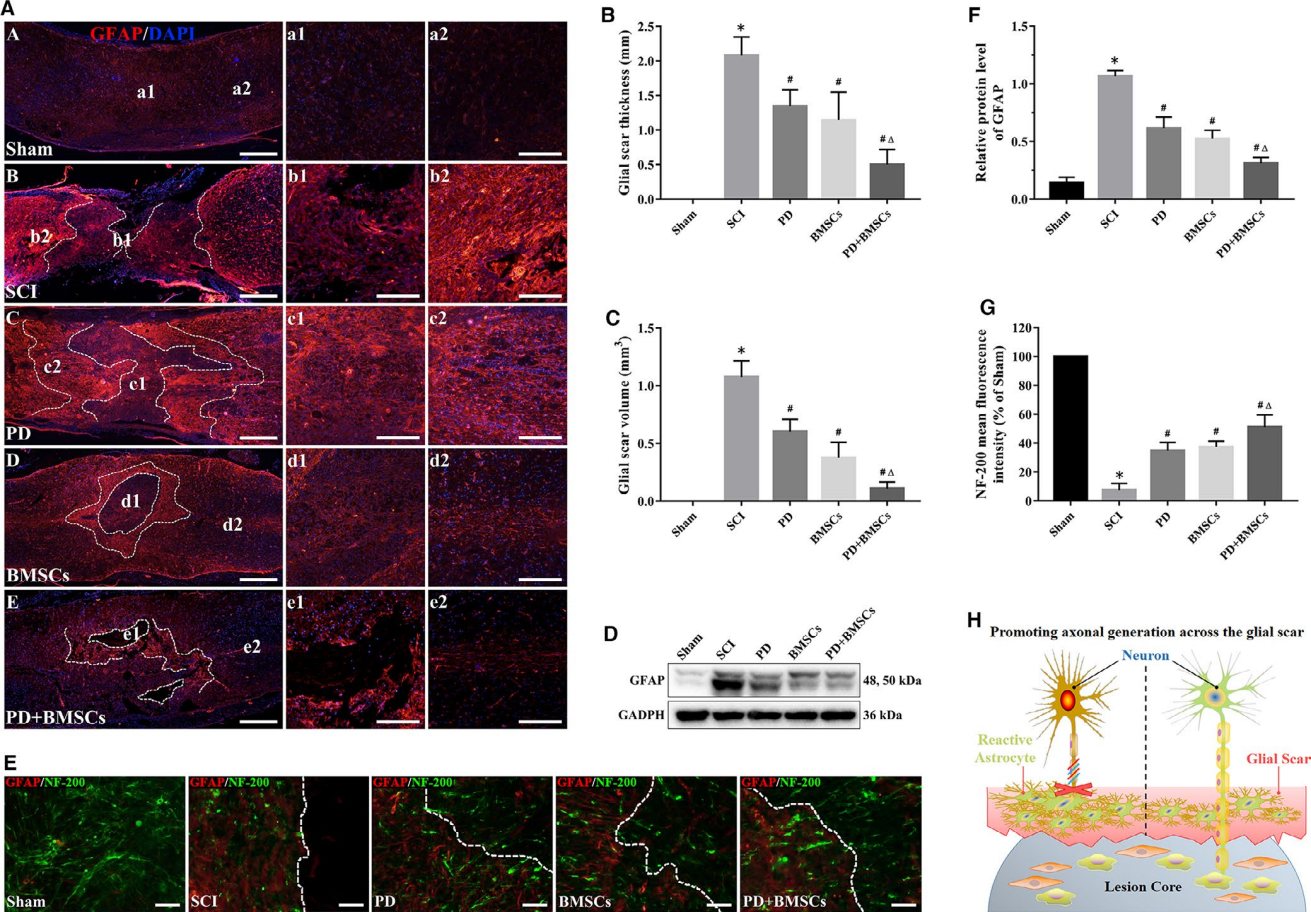
PD and BMSCs attenuated glial scar formation and promoted axonal generation across the glial scar. A, Immunofluorescence images of the spinal cord show reactive astrogliosis and glial scar formation (scale bar = 500 and 200 μm). (B, C) Quantitative analysis of thickness and volume of glial scar. (D, E) Relative GFAP protein levels in each group. F, Representative images showing the astrocytic fronts (dashed lines) and neurofilaments (NF‐200) on spinal cord sections (scale bar = 50 μm). G, Quantitative analysis of the NF‐200 fluorescence intensity. H, Schematic of PD + BMSC‐mediated axonal generation across the glial scar. *P < .05 vs sham; ^#^ *P* < .05 vs SCI; and ^Δ^ *P* < .05 vs PD and BMSCs

Although the transplanted BMSCs can migrate to the injured area and differentiate into neuron‐like cells, their therapeutic effects are often impeded by the formation of glial scars around the lesions. GFAP + glial scars were detected at the epicentre of the injured spinal cord and were significantly weakened in the dual‐treated mice at 28 dpi compared with the mice subjected to either PD or the BMSCs (Figure 6A). The thickness and volume of glial scar respectively decreased from 1.71 ± 0.60 mm and 0.38 ± 0.13 mm^3^ in the untreated mice to 0.75 ± 0.33 mm and 0.11 ± 0.06 mm^3^ in the PD + BMSC group (Figure 6B,C). Furthermore, the total levels of the GFAP protein also decreased at the injured sites following the combination therapy (Figure 6D,F). Post‐SCI repair depends on whether the regenerated axons of the rostral spinal cord stump can pass through the glial scar. We detected a complete absence of neurofilaments at the epicentre of the injured spinal cord in the untreated mice (Figure 6F), whereas the combination therapy elicited significant growth of the NF‐200+ neurofilaments beyond the glial scar compared with the respective monotherapies (Figure 6G). Taken together, PD augmented the BMSC‐driven axonal regeneration in the injured spinal cord and weakened glial scars to accelerate SCI repair (Figure 6H).

### 3.6 PD and BMSCs improved locomotor function

The findings so far indicated that PD encouraged neural differentiation of transplanted BMSCs, increased axonal rehabilitation and attenuated glial scar formation in mice with SCI. To determine whether these effects translated into motor function recovery, we subjected the animals to the BBB rating scale and inclined plane tests. All mice showed complete hindlimb paraplegia immediately after SCI, which was partially restored over time (Figure S5A). In contrast, the sham‐operated mice exhibited no locomotor impairment during the observation period. The combination therapy group showed maximum locomotor recovery in terms of the BBB and inclined plane test scores compared with the untreated and PD/BMSC‐treated groups (Figure S5B,C). In addition, the hindlimb weight‐bearing capacity and gait co‐ordination of the PD and BMSC‐treated mice improved significantly, and almost achieved the functional levels of the sham group. We also analysed the electrophysiological recovery of the differentially treated mice at 28 dpi. SCI significantly decreased the SCEP amplitudes (0.25 ± 0.08 mv) and prolonged the SCEP latencies (10.63 ± 0.91 ms) compared with those in the sham group. However, the combination of PD and BMSCs significantly increased the SCEP amplitudes (0.73 ± 0.08 mv) and shortened the SCEP latencies (5.45 ± 0.57 ms) compared with those in the untreated mice (Figure S5D‐F).

### 3.7 PD promoted neuronal differentiation of BMSCs via Nrf2 activation

**FIGURE 7:**
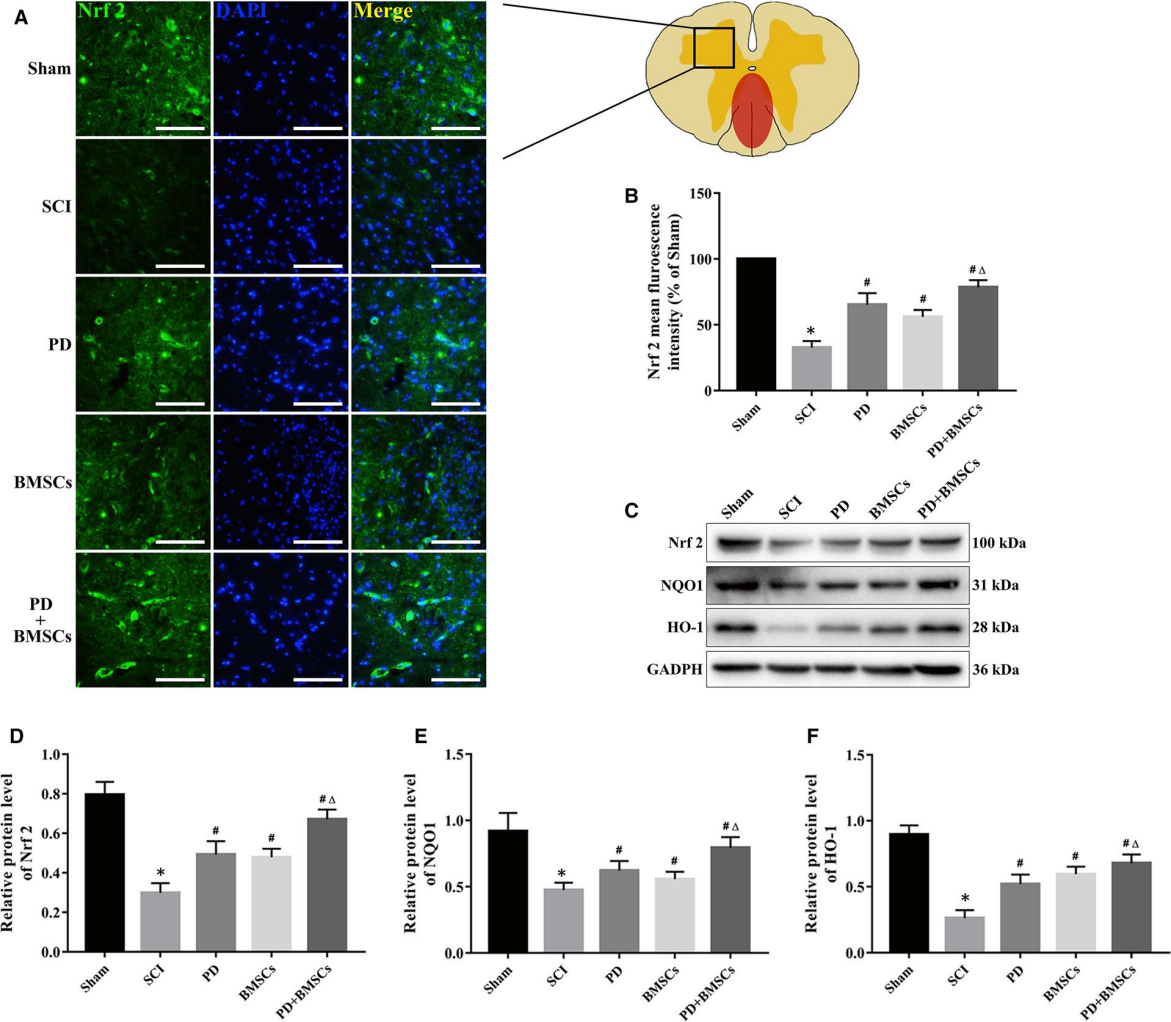
PD‐induced neuronal differentiation of BMSCs in vivo by activating the Nrf2 signalling pathway. A, Fluorescence images of spinal cord showing in situ expression of Nrf2 (scale bar = 100 μm). B, Quantitative analysis of the fluorescence intensities. C, Immunoblot showing Nrf2, NQO1 and HO‐1 levels in the injured spinal cord at 28 dpi. D‐F, Quantification of relative protein levels. *P < .05 vs sham; ^#^ *P* < .05 vs SCI; and ^Δ^ *P* < .05 vs PD and BMSCs

Nrf2 up‐regulation and nuclear translocation are critical for neuronal cell differentiation. To determine whether PD‐induced neural differentiation of BMSCs involved Nrf2 signalling, we analysed the expression levels of Nrf2 and its downstream targets in the neuron‐like differentiated cells. Although neural induction in vitro led to increased levels of Nrf2, NQO1 and HO‐1 independent of PD, the latter further augmented their expression (Figure S6A‐C). Consistent with this, the proportion of Nrf2‐positive cells was reduced in spinal cord lesions after SCI, but increased after the respective treatments, particularly in the PD+BMSC group (Figure 7A,B). Furthermore, the spinal cord tissues showed increased levels of NQO1 and HO‐1 as well after the combination treatment compared with the other groups (Figure 7C‐F).

To further explore the relationship between PD‐Nrf2 axis and the neurogenic potential of BMSCs, we inhibited Nrf2 during the neural induction of BMSCs using brusatol. As shown in Figure S6D‐K, pharmacological inhibition of Nrf2 significantly down‐regulated MAP‐2, NeuN, NF‐M and NSE. Taken together, PD augments the neuronal differentiation of BMSCs in the injured spinal cord and aids in functional recovery of the BMSC‐treated mice via the Nrf2 signalling pathway.

## 4 DISCUSSION

Axonal disintegration and neuronal apoptosis after SCI are the major causes of hindlimb motor deficits.^25^ Therefore, in order to restore spinal cord function, it is essential to boost axonal regeneration and neuronal restoration. Stem cell transplantation has gained considerable attention in recent years as a novel therapeutic strategy against SCI.^26^, ^27^ Due to the limited number of resident nerve cells,^28^ BMSCs are a viable source of donor neuronal cells for regenerative repair.^29^ However, the transplanted BMSCs have a limited capacity of differentiating into neuron‐like cells in situ and therefore primarily regulate the microenvironment through paracrine mechanisms,^30^ rather than directly giving rise to new axons. Therefore, there is an urgent need to enhance the neuronal differentiation of BMSCs, as well as elucidate the mechanisms through which these cells can improve SCI‐induced motor deficits. To this end, we established a murine model of SCI and transplanted them with BMSCs with or without the neuroprotective agent PD.

PD augmented the neural differentiation of murine BMSCs both in vitro and in vivo, as indicated by the increased expression levels of neuronal markers such as MAP‐2, NeuN, NF‐M and NSE. Furthermore, the neuron‐like cells arising from the PD‐treated BMSCs following neural induction expressed high levels of ChAT, indicating that these cells were likely cholinergic. The cholinergic motor neurons activate skeletal muscles and therefore are pivotal for locomotion and behavioural responses.^31^ Thus, PD can enhance the therapeutic effect of BMSCs by facilitating their differentiation into cholinergic motor neurons. PD and other components of traditional Chinese medicine are more stable and non‐toxic than antioxidants, neurotrophic factors and physical co‐culture for neural induction.^32^, ^33^, ^34^ It is widely regarded that both PD and BMSCs possess notable anti‐inflammatory and neuroprotective activities that may ameliorate the devastating second injury resulting from SCI.^35^, ^36^ However, the results of our in vivo supplemental experiments showed no significant differences in anti‐inflammatory, antioxidant or neuroprotective effects among the three treatment groups, suggesting that PD plays a therapeutic role in SCI mice by promoting neural differentiation of BMSCs rather than by neuroprotection. Recent evidence has also indicated that the inhibition of axonal regeneration is mainly due to excessive glial scar tissue formation^37^; furthermore, preventing the proliferation of scar‐forming astrocytes could effectively improve functional deficiency after SCI.^38^ Our results showed that the physical barrier, such as reactive astroglial proliferation and glial scar formation, was markedly reduced after PD+BMSC treatment. Moreover, more NF‐200–positive neurofilaments were detected at the lesion site after SCI. Animal studies have demonstrated that both PD and transplanted BMSCs modify the inflammatory immune microenvironment in the acute setting and reduce the effects of the inhibitory scar tissue in the subacute/chronic setting to provide a permissive environment for neural differentiation and axonal extension.^39^, ^40^, ^41^

However, as PD alone cannot initiate the differentiation of BMSCs, it is possible that it likely sensitizes the BMSCs to other neural induction factors by targeting certain intrinsic, neurogenic signalling pathways. The molecular mechanisms controlling neuronal differentiation are highly complex and involve multiple signalling pathways. Neurite outgrowth requires precise regulation of actin filaments, microtubules and intermediate filaments, the major structural proteins that form the cytoskeleton.^42^ In addition, multiple pathways converge to alter the expression of cytoskeleton proteins (including MAPs and neurofilaments), and to achieve precise regulation of cytoskeletal dynamics to accommodate neurite outgrowth.^43^, ^44^, ^45^ Previous studies have shown the involvement of the Notch1, PI3K/AKT, MAPKs and Wnt pathways in the neuronal differentiation of BMSCs.^46^, ^47^, ^48^ We previously reported that PD up‐regulated Nrf2 and its target genes in BMSCs in a dose‐dependent manner^14^ and Nrf2 induction by PD increased neurite outgrowth and up‐regulated MAP2, NeuN, NF‐M and NSE. Furthermore, the neurite outgrowths induced in the presence of PD were longer than those in its absence. In agreement with our in vivo findings, two well‐recognized neural inducers, 12‐O‐tetradecanoylphorbol‐13‐acetate and retinoic acid, promoted neuronal differentiation of SH‐SY5Y cells by up‐regulating Nrf2.^19^

Nrf2 is the master regulator of multiple antioxidant genes and also influences cell proliferation, migration, inflammation, neurite outgrowth and differentiation.^49^, ^50^, ^51^, ^52^ Targeted inhibition of Nrf2 in the BMSCs blocked neuronal induction in the presence of PD in our study, while overexpression of Nrf2 had the opposite effect on SH‐SY5Y cells.^19^ Similarly, exogenous expression of Nrf2 enhanced the differentiation potential of neural progenitor cells (NPCs) isolated from both wild‐type and Nrf2‐null mice.^49^ To elucidate the effect of the PD‐Nrf2 axis on the neuronal differentiation of BMSCs, brusatol was used to block the Nrf2 pathway, which was also considered to have a similar effect to lentivirus‐mediated transfection.^53^, ^54^ Similar to the studies mentioned above, we found that the effect of PD‐mediated up‐regulation of Nrf2, thereby promoting neuronal differentiation of BMSCs, was significantly inhibited. Furthermore, activation of the Nrf2 pathway promoted neurotrophin‐induced axon growth from PC12 cells.^55^, ^56^ Interestingly, PD restored HO‐1 levels in the differentiating BMSCs at the later stages,^57^, ^58^ which was correlated with improved viability and greater resistance to oxidative stress. The early molecular events of CNS trauma are increased oxidative stress and mitochondrial dysfunction, which are the key factors driving neurodegeneration.^59^, ^60^ Recent studies show that neural differentiation increases resistance to active lipid or reactive oxygen species.^61^, ^62^ These findings corroborate our hypothesis that PD could enhance the neuronal differentiation of BMSCs and improve survival of the neuron‐like cells in vivo, making it a promising adjuvant for regenerative therapy in SCI.

In summary, PD promotes BMSC differentiation into neuron‐like cells in vivo and in vitro through Nrf2 activation. Supplementing BMSC transplantation with PD can significantly enhance axonal rehabilitation and attenuate glial scar formation during SCI, indicating a potential therapeutic strategy.

## CONFLICT OF INTEREST

The authors declare no conflicts of interest.

## AUTHOR CONTRIBUTIONS

LDK and HYH did the conception and design of the research. ZJH, LX and LD performed the experiments. HY and CSD analysed the data. XZF and LJY prepared the figures. ZJH, LX and LD performed the drafting of the article. All authors read and approved the final manuscript.

## Supporting information

Supplementary Material

## Data Availability

The data sets used to support the findings of this study are available from the corresponding author upon request.
