# Supplementary material for: Polydatin promotes the neuronal differentiation of bone marrow mesenchymal stem cells in vitro and in vivo: Involvement of Nrf2 signalling pathway
Source: J Cell Mol Med. 2020 Apr 16;24(9):5317–29. doi: 10.1111/jcmm.15187 (PMC7205798; doi:10.1111/jcmm.15187)
Supplement: Supplementary file 1 — Supplementary Material [file JCMM-24-5317-s001.docx]

**Supporting information**

Polydatin promotes the neuronal differentiation of bone-marrow mesenchymal stem cells *in vitro* and *in vivo*: Involvement of Nrf2 signaling pathway

Running title: Polydatin promotes BMSC neuronal differentiation.

Jiheng Zhan^12#^, Xing Li^23#^, Dan Luo^23#^, Yu Hou^13^, Yonghui Hou^23^, Shudong Chen^3^, Zhifeng Xiao^3^, Jiyao Luan^12^, Dingkun Lin^3*^

^1^Second Clinical College, Guangzhou University of Chinese Medicine, Guangzhou, 510405, China.

^2^Lingnan Medical Research Center, Guangzhou University of Chinese Medicine, Guangzhou, 510405, China.

^3^Department of Spine Surgery, The Second Affiliated Hospital of Guangzhou University of Chinese Medicine, Guangzhou, 510120, China.

^#^ Jiheng Zhan, Xing Li, and Dan Luo contributed equally to this work.

*Correspondence to: Prof. Dingkun Lin (lindingkun@126.com). Phone: +86-02081887233, Fax: +86-02081887233. Department of Spine Surgery, The Second Affiliated Hospital of Guangzhou University of Chinese Medicine, No. 111 Dade Road, Yuexiu District, Guangzhou, 510120, China.

**Supplemental table**

**Table S1. Primers used for targets amplification in this study**

| Name | Primer | Accession number | Sequence(5’-3’) |
| --- | --- | --- | --- |
| MAP-2 | Forward | NM_001039934 | TGTGCTGTGTGCTCCAAGTT |
|  | Reverse |  | AGTGCTTCCCAATCAGTGCT |
| NeuN | Forward | XM_006533726 | TAACTAGCCCCACTGTTGTACC |
|  | Reverse |  | ATTGCCTCTGAGCTGTCAAGA |
| NF-M | Forward | NM_008691 | CGACAGCCCTCAGTCACAAT |
|  | Reverse |  | CGATGGCTGTGAGGGTTTCT |
| NSE | Forward | NM_013509 | CTCGAGGAGATCCCAGCCA |
|  | Reverse |  | CTCATAGATGCCGGTGGAGG |
| Nrf 2 | Forward | NM_010902 | CCCAGCAGGACATGGATTTGA |
|  | Reverse |  | AGCTCATAGTCCTTCTGTCGC |
| NQO1 | Forward | NM_008706 | TCTCTGGCCGATTCAGAGTG |
|  | Reverse |  | CCAGACGGTTTCCAGACGTT |
| HO-1 | Forward | NM_010442 | GCTAGCCTGGTGCAAGATACT |
|  | Reverse |  | AAGCTGAGAGTGAGGACCCA |
| *β*-Actin | Forward | NM_007393 | TGAGCTGCGTTTTACACCCT |
|  | Reverse |  | GCCTTCACCGTTCCAGTTTT |

**Supplemental figures and figure legends**

**Figure S1.**

**
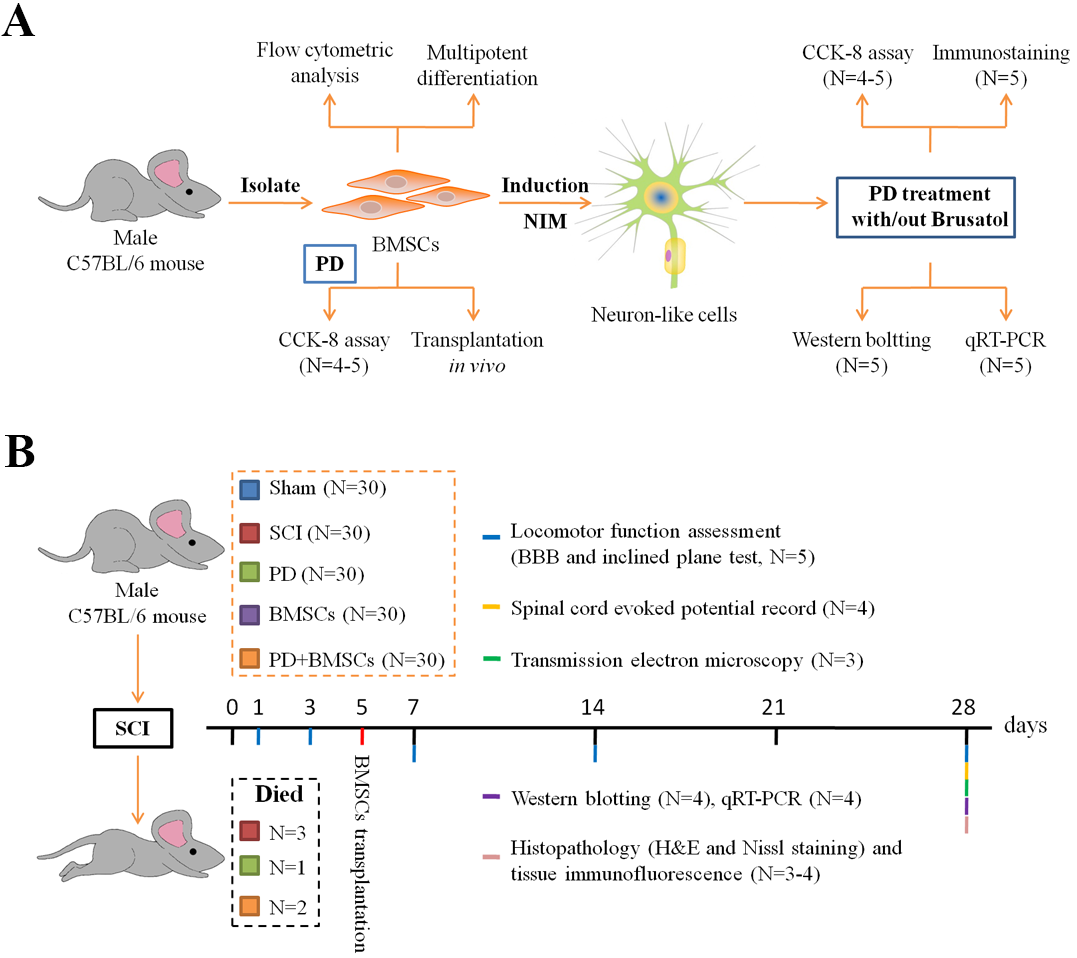
**

**Figure S1.** Flow-chart schematic describing the experimental design of the study. (A) Cell experiment. (B) Murine spinal cord surgery experiment.

**Figure S2.**

**
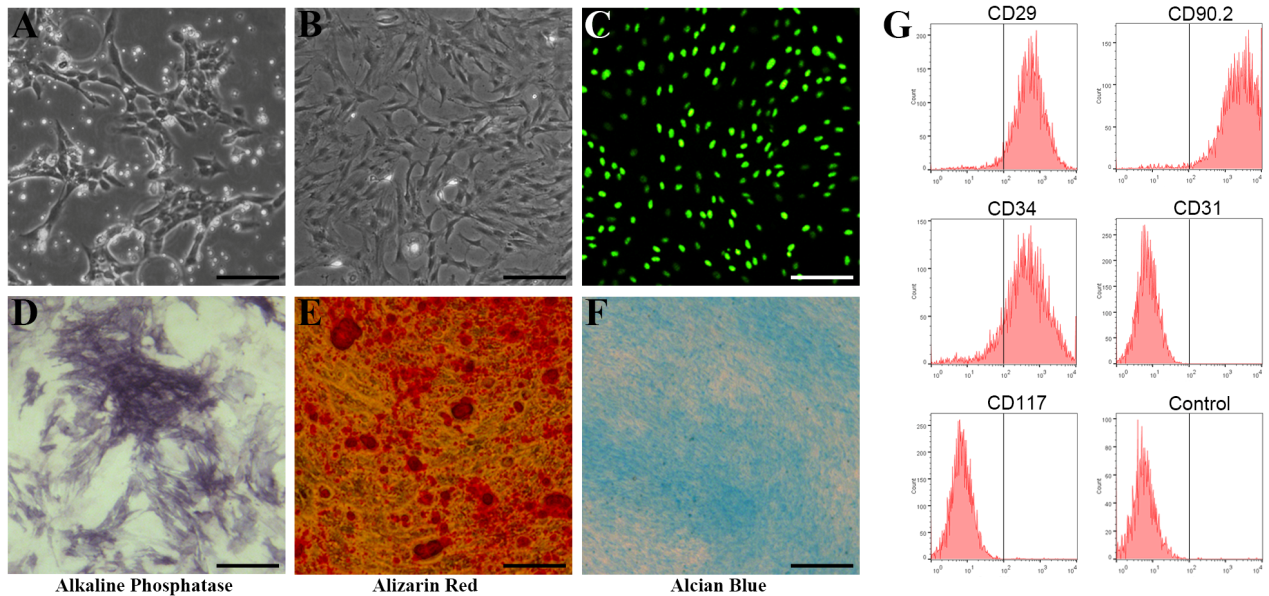
**

**Figure S2.** **Phenotypic and functional characterization of isolated BMSCs.** Representative images of BMSCs at passages (A) 0 and (B) 3. (C) BrdU-labeled BMSCs prior to transplantation. (D-F) BMSCs differentiated into osteogenic and chondrogenic lineages. (G) Flow cytometric analysis of the cultured BMSCs showing CD29^+^, CD90.2^+^, CD31^–^, CD117^–^ and CD34^+^ immunophenotype. Scale bar = 20μm.

**Figure S3.**


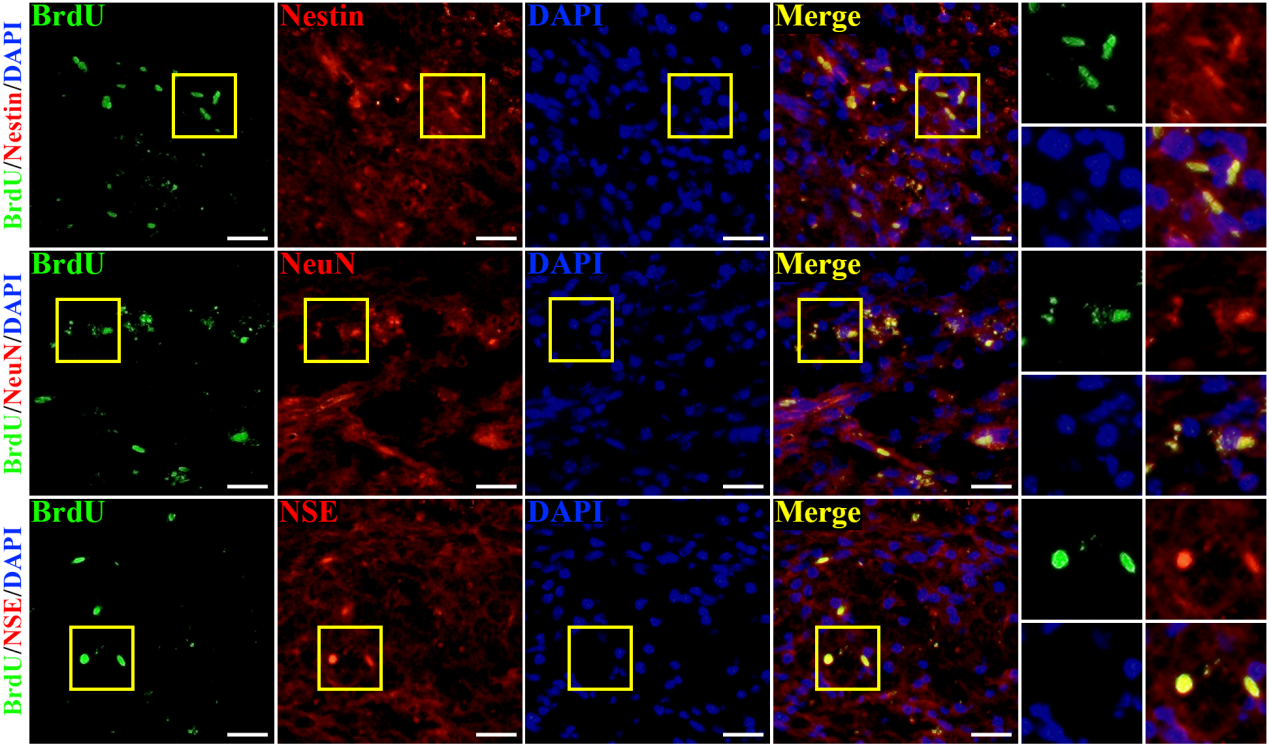


**Figure S3. BrdU-labeled BMSCs *in vivo* expressed neuronal markers.** Neuronal-like differentiation of the BMSCs transplanted *in vivo* in the BMSCs group. Inset squares indicate the co-localization of BrdU with Nestin, NeuN or NSE. Scale bar = 20μm.

**Figure S4.**

**
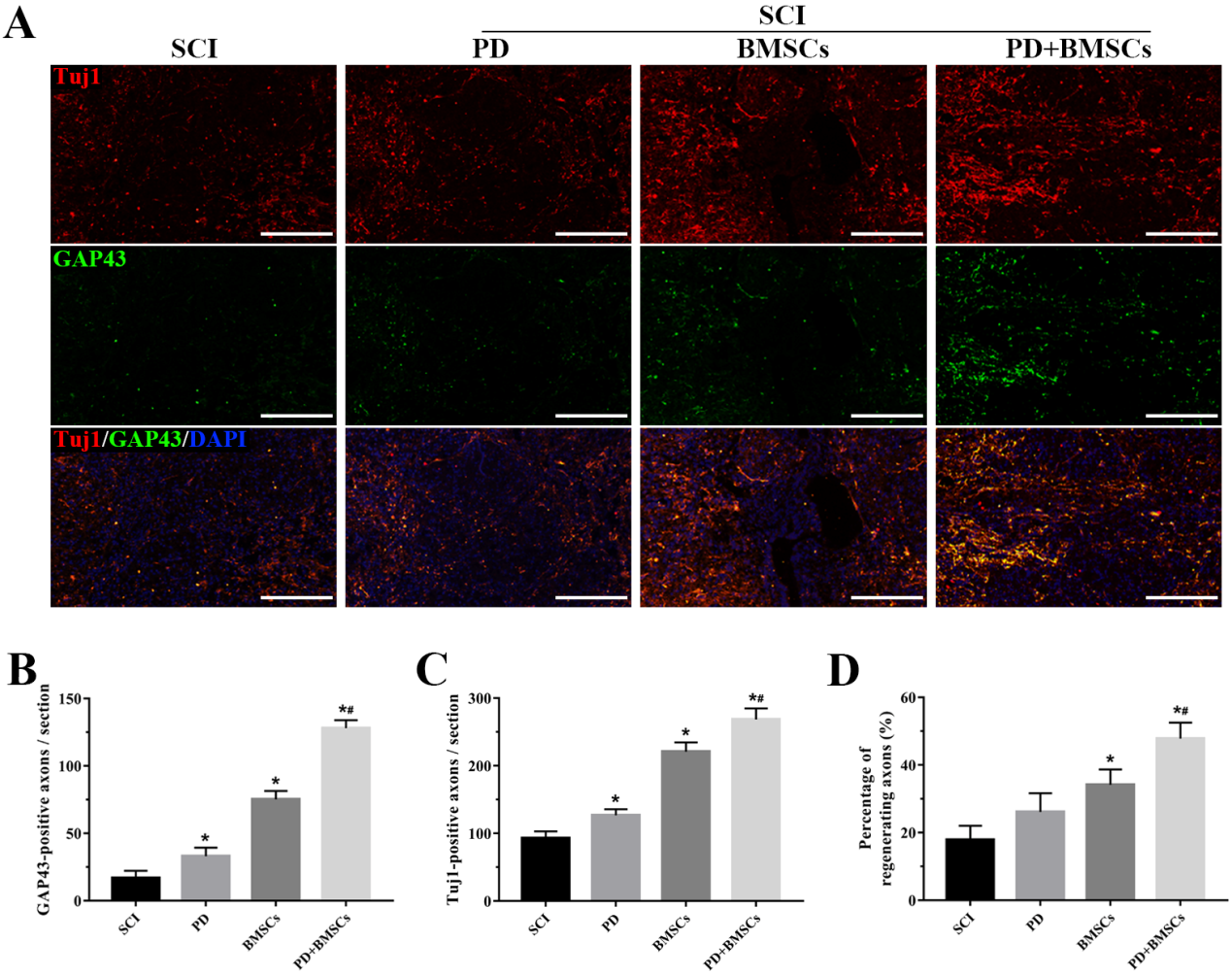
**

**Figure S4. PD and BMSCs promoted axonal regeneration.** (A) Representative immunofluorescence images showing Tuj1 (red) and GAP43 (green) co-expressing axons. The number of (B) GAP43+ and (C) Tuj1+ axons, and (D) the percentage of regenerating axons. Scale bar = 200μm.

**Figure S5.**


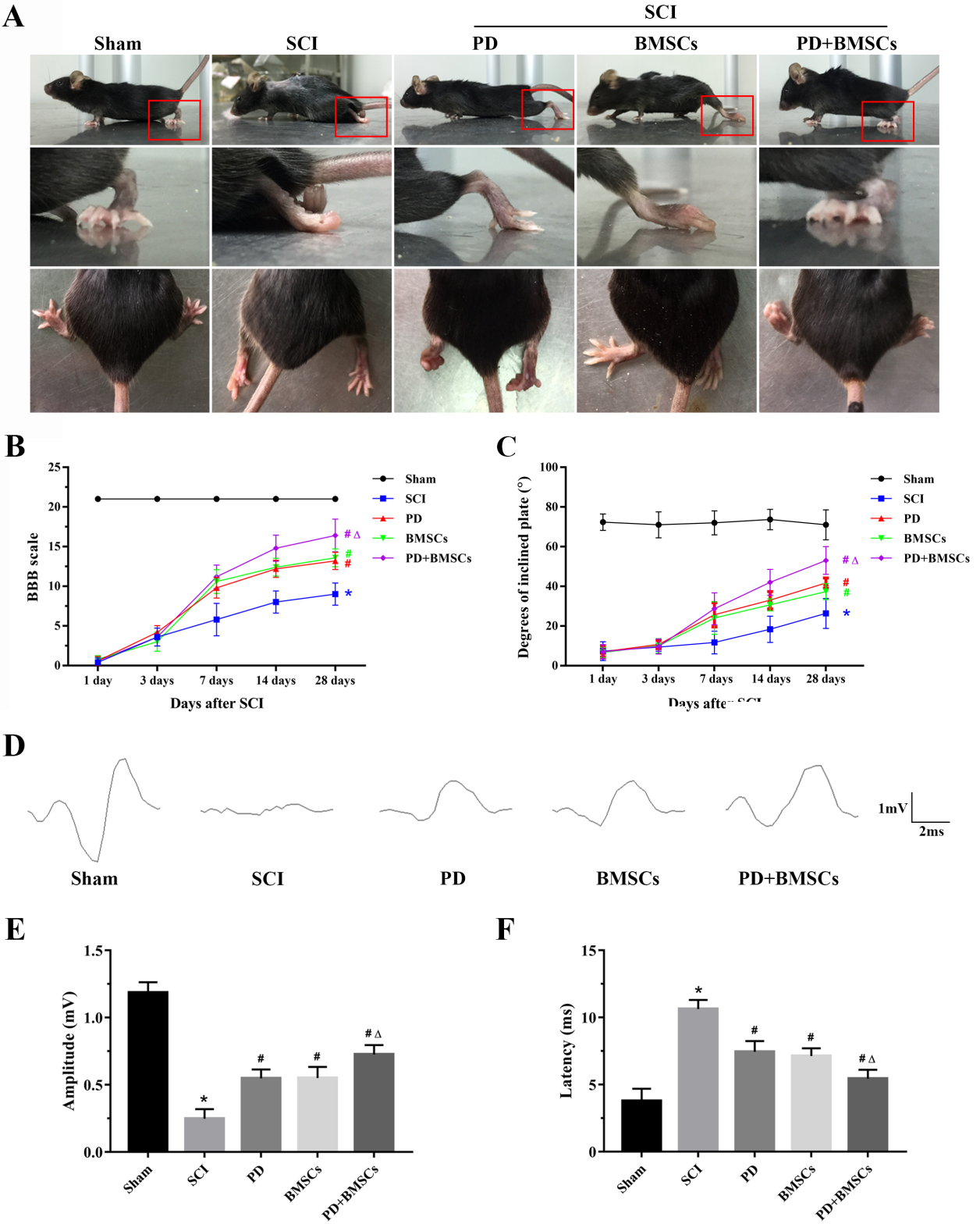


**Figure S5.** **PD and BMSCs** **improved the locomotor function recovery after SCI.** (A) Representative images showing hindlimb motion in different groups. (B) The BBB locomotion scores and (C) the degrees of inclined plane in different groups. (D) Typical SCEP response from each group. (E, F) Quantification analysis of the amplitude and latency of SCEP signals. **P*< 0.05 vs. sham; #*P*< 0.05 vs. SCI; Δ*P*< 0.05 vs. PD and BMSCs.

**Figure S6.**


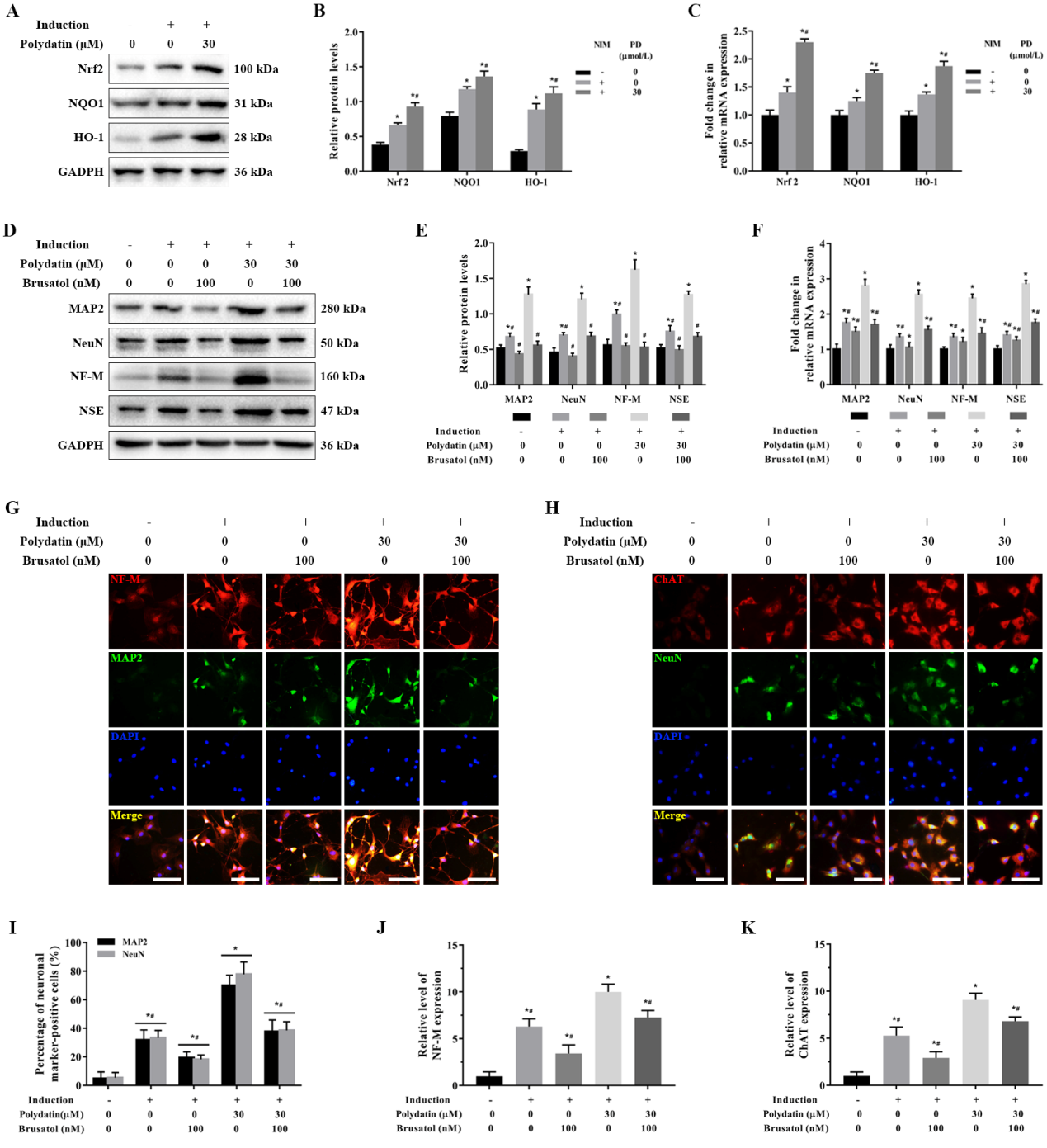


**Figure S6. PD activated the Nrf2 pathway during neuronal differentiation of BMSCs.** (A, B) Relative Nrf2, NQO1 and HO-1 protein levels in each group. (C) Relative mRNA levels of the above factors. **P*< 0.05 vs. CT; #*P*< 0.05 vs. standard induction (NIM) group. (D) Immunoblot showing MAP-2, NeuN, NF-M and NSE levels in the differentiated cells with/out brusatol, and (E) quantification of the relative protein expression levels. (F) Fold changes in the mRNAs compared to the CT group. (G, H) Representative immunofluorescence images showing BMSCs expressing neuronal markers, scale bar = 100μm. (I-K) The number of marker-positive cells relative to the total BMSCs. **P*< 0.05 vs. CT; #*P*< 0.05 vs. PD.
